# Home-Enclosure-Based Behavioral and Wireless Neural Recording Setup for Unrestrained Rhesus Macaques

**DOI:** 10.1523/ENEURO.0285-22.2022

**Published:** 2023-01-09

**Authors:** Laura Hansmeyer, Pinar Yurt, Naubahar Agha, Attila Trunk, Michael Berger, Antonino Calapai, Stefan Treue, Alexander Gail

**Affiliations:** 1 Cognitive Neuroscience Laboratory, German Primate Center, 37077 Göttingen, Germany; 2 Bernstein Center for Computational Neuroscience, University of Göttingen, 37073 Göttingen, Germany; 3 Leibniz ScienceCampus Primate Cognition, 37077 Göttingen, Germany; 4 Georg-August University School of Science, 37073 Göttingen, Germany; 5 Göttingen Graduate Center for Neurosciences, Biophysics, and Molecular Biosciences, University of Göttingen, 37073 Göttingen, Germany; 6 Faculty for Biology and Psychology, University of Göttingen, 37073 Göttingen, Germany; 7 Laboratory of Neural Systems, The Rockefeller University, New York, NY 10065

**Keywords:** cage-based testing, wireless neural recordings

## Abstract

Electrophysiological studies with behaving nonhuman primates often require the separation of animals from their social group as well as partial movement restraint to perform well-controlled experiments. When the research goal per se does not mandate constraining the animals’ movements, there are often still experimental needs imposed by tethered data acquisition. Recent technological advances meanwhile allow wireless neurophysiological recordings at high band-width in limited-size enclosures. Here, we demonstrate wireless neural recordings at single unit resolution from unrestrained rhesus macaques while they performed self-paced, structured visuomotor tasks on our custom-built, stand-alone touchscreen system [eXperimental Behavioral Instrument (XBI)] in their home environment. We were able to successfully characterize neural tuning to task parameters, such as visuo-spatial selectivity during movement planning and execution, as expected from existing findings obtained via setup-based neurophysiology recordings. We conclude that when movement restraint and/or a highly controlled, insulated environment are not necessary for scientific reasons, cage-based wireless neural recordings are a viable option. We propose an approach that allows the animals to engage in a self-paced manner with our XBI device, both for fully automatized training and cognitive testing, as well as neural data acquisition in their familiar environment, maintaining auditory and sometimes visual contact with their conspecifics.

## Significance Statement

Cage-based testing systems have previously been shown to be highly useful in cognitive assessment of nonhuman primates. These systems allow animals to engage with the task/system in an unrestrained and self-paced manner. We expanded the capabilities of our own cage-based testing device by combining cognitive testing with wireless neural recordings in the animals’ home environment, in an upscalable approach. When neither movement constraints nor specialized equipment are scientifically necessary, our approach allows for the combination of cognitive testing with intracranial electrophysiology without removing the animal from its home environment, potentially improving animal well-being.

## Introduction

System-level neurophysiological studies in awake behaving animals usually require partial movement restraint and separation from the social group. Animals are typically transferred to an isolated setup to achieve a highly controlled experimental setting with minimal distractions which is equipped with a recording apparatus to acquire behavioral and neural activity. Depending on the scientific research question and the way behavioral and physiological data are measured, it is necessary to restrain body and head movements to a variable degree and to control eye movements, which is achieved by sitting in a dedicated transport device (monkey chair). For some research questions neither movement restraint nor social isolation are essential. Here, cage-based, wireless recording techniques might represent a valuable alternative to traditional setup-based recordings. By avoiding movement restraints and social isolation over the course of several hours during an experiment, home-cage based approaches have the potential to improve animal welfare.

A variety of experimental settings to perform electrophysiological recordings with largely unrestrained nonhuman primates have been described in recent years. While some studies focused on untethered recordings from freely moving rhesus macaques in dedicated setups (Foster et al., 2014; Schwarz et al., 2014; Yin et al., 2014; Fernandez-Leon et al., 2015; Capogrosso et al., 2016; Libedinsky et al., 2016; Rajangam et al., 2016; Berger et al., 2020; Milton et al., 2020; Mao et al., 2021), others performed untethered recordings in the animals’ home environment without cognitive tasks (Jackson et al., 2006, 2007; Chestek et al., 2009; Borton et al., 2013; Xu et al., 2019; Zhou et al., 2019). Here, we present a technique where a wireless recording system is coupled with our cage-based, stand-alone, cognitive testing system [eXperimental Behavioral Instrument (XBI); Calapai et al., 2017; Berger et al., 2018]. This system allows the animals to engage in a self-paced manner with the same device, both for fully automatized training (Berger et al., 2018; Calapai et al., 2022) as well as neural data acquisition in their familiar environment, maintaining auditory and sometimes visual contact with their conspecifics.

Automated cognitive testing and training animals in their home environment has already been established in different labs (Rumbaugh et al., 1989; Andrews and Rosenblum, 1994; Crofts et al., 1999; Evans et al., 2008; Fagot and Paleressompoulle, 2009; Rodriguez et al., 2011; Takemoto et al., 2011; Gazes et al., 2013; Calapai et al., 2017; Claidière et al., 2017; Curry et al., 2017; Tulip et al., 2017; Berger et al., 2018; Butler and Kennerley, 2019; Jacob et al., 2021; Sacchetti et al., 2021; Cabrera-Moreno et al., 2022). In this study we expanded the capabilities of our XBI system in the home-environment of the monkeys by combining it with a wireless neural acquisition system capable of recording up to 192 channels of high bandwidth signals from chronically implanted intracortical microelectrodes. We simultaneously recorded single unit activity from three cortical areas in the fronto-parietal reach network of two rhesus macaques performing a visually instructed, goal-directed, memory-guided reach task in their home environment. In summary, we present an integrated approach allowing wireless neural recordings combined with cognitive testing while the animals are kept in their home environments.

## Materials and Methods

### Study subjects, surgical implants, and housing conditions

We conducted the study on two adult male rhesus macaques (*Macaca mulatta*, K and H, seven and 12 years of age). The animals were group-housed with other macaque monkeys in facilities of the German Primate Center in Göttingen, Germany, in accordance with all applicable German and European regulations. The facilities provide the animals with an enriched environment (including multitude of toys and wooden structures and natural as well as artificial light, exceeding the size requirements of the European regulations, including access to outdoor space).

Both animals participated in previous cognitive experiments including electrophysiological recordings. As part of these previous studies, monkey K was implanted with six 32-channel floating microelectrode arrays (Microprobes for Life Sciences) with custom electrode length in the primary motor cortex (M1), the dorsal premotor cortex (PMd), and the parietal reach region (PRR) of the right hemisphere. For the purpose of the current study, we implanted monkey H with the same microelectrode array system as monkey K, in area M1, PMd, and supplementary motor area (SMA) of the left cerebral hemisphere. The surgery protocol is described in a previous publication (Berger et al., 2020). Using the same surgery protocol, monkey H was implanted with the FMAs and the components of the wireless chamber system in a single sterile surgery under general anesthesia and analgesia. Array connectors as well as recording equipment were protected by custom-designed implants (Berger et al., 2020; Ahmed et al., 2022). While chair-seating the animals was not needed for the recording sessions per se, animals were trained to sit in a primate chair for mounting of recording equipment and implant care. Both monkeys were housed in social groups of two (animal H) or three (animal K) males, respectively.

### Ethical statement

Research with nonhuman primates represents a small but indispensable component of neuroscience research. The scientists in this study are aware and are committed to the great responsibility they have in ensuring the best possible science with the least possible harm to the animals (Roelfsema and Treue, 2014; Treue and Lemon, 2022). We have established a comprehensive set of measures to ensure that the severity of our experimental procedures falls into the category of mild to moderate, according to the severity categorization of Annex VIII of the European Union’s directive 2010/63/EU on the protection of animals used for scientific purposes (see also Pfefferle et al., 2018).

All procedures comply with the German Law and the European Directive 2010/63/EU and have been approved by regional authorities [Niedersächsisches Landesamt für Verbraucherschutz und Lebensmittelsicherheit (LAVES)] under permit number 33.19–42502-04–18/2823.

### Experimental setup

**Figure 1.**
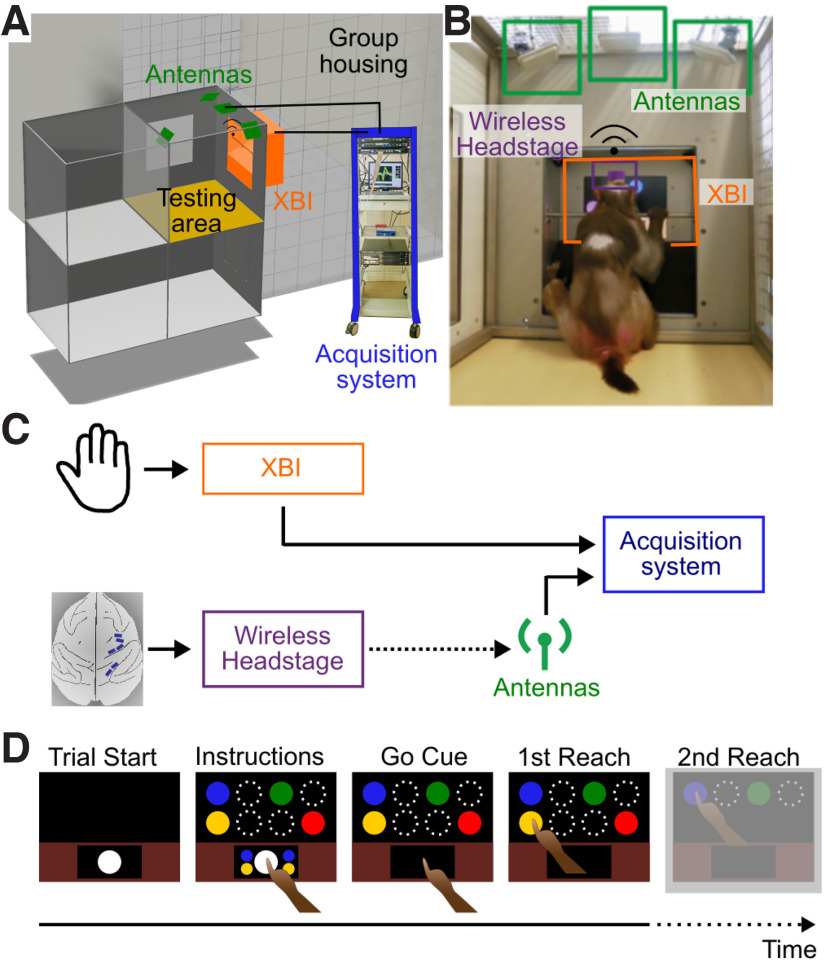
Experimental setup and task design. ***A***, Schematic of the experimental setting. The recording sessions take place inside the testing area (shown in yellow) next to the group housing. Four antennas (shown in green) are installed at the ceiling of the testing area. The experimenter has access to the XBI (shown in orange) from the back. The data acquisition system is shown in blue. ***B***, Animal K working on the XBI inside the testing area. The wireless head stages are protected with a cap (shown in purple). ***C***, Data flow during recordings. Neural signals are collected, digitized, and sent wirelessly to the receiving antennas. At the same time the animal interacts through a touch screen with the XBI. Neural signals are sent to the acquisition system together with time stamps for trial starts for offline synchronization with behavioral data. ***D***, Behavioral task design. The animals are required to perform two consecutive reaches to a first target in the lower row and a second target in the upper row. Upon touching the starting position, two possible targets appear in both rows together with two color cues next to the starting position, indicating the correct target in each row. Circles with white dashed lines show additional possible reach target (not visible to the animal). Reach target and colors are randomized trial-wise. The second reach is greyed out as analysis in this study only includes times until touching the first target.

During each experimental session, animals autonomously operated an updated version of our previously reported touchscreen-based, stand-alone XBI device for cage-based cognitive testing (Calapai et al., 2017; Berger et al., 2018). The XBI [50 × 57 × 31.5 cm (W × H × D)] was attached to one side of the testing area (1 m^3^, highlighted in yellow) directly adjacent to and accessible from the animals’ group housing enclosure (Fig. 1*A*). During recording sessions, the animals were kept alone in the testing area with auditory and visual contact with its members of the housing group or other groups in the same animal facility.

The behavioral task was presented and controlled by the open-source software MWorks (version 0.8; http://mworks-project.org/). The XBI used in this study was controlled by a laptop computer (Apple MacBook Air 1.6 GHz Intel Core i5, 8GB RAM, macOS High Sierra version 10.13.6) and a custom-made microcontroller (based on a Teensy 3.5) to provide visual stimuli and register manual responses by the animal via the touchscreen (15 inches, ELO 1537L Securetouch) and to control the reward system, consisting of two separate peristaltic pumps (Verderflex OEM-Schlauchpumpe M025 DC, 10–30 V, 6.5 W) and a custom-made multi-fluid mouth piece to deliver two types of reward jointly or independently.

### Neural recordings

Neural activity from all six 32-channel arrays was acquired simultaneously at a 30-kHz sampling rate per channel, digitized at 16-bit amplitude resolution, and wirelessly transmitted with 12-bit resolution at 3.05 GHz and 3.375 GHz, respectively, carrier frequency via two separate head stages (96-channel Exilis Headstage, Blackrock Microsystems LCC) from both monkeys. We have decided to use the smaller and lighter head stages designed for small laboratory animals as this allowed us to work with two head stages in parallel being able to record from 192 electrodes simultaneously. To prevent interference, we added a metal film as a separator between the head stages which was connected to the ground of one head stage. Because of leakage, we lost the arrays in PMd and SMA in monkey H and only report on the data from M1. The transmitted signal was captured by four antennas strategically located at the top of the testing area to best cover the area directly in front of the XBI touchscreen and reward mouth piece (see Fig. 1*A*,*B*) and connected to two receivers (one for each head stage). From a previous study (Berger et al., 2020) we know that eight receiving antennas are needed to cover a space which is twice as big as the testing area in this study. We, therefore, opted for four receiving antennas as experience shows that the animals keep a stable head position when working on the XBI because of the centrally located mouth piece to deliver reward. Signals were then processed by a 256-channel Cereplex Direct data acquisition system (Blackrock Microsystems LLC).

The behavioral data acquired through the XBI touchscreen are synchronized offline with the neural signals after digitization and acquisition by the Cereplex system. For this, the trial number is sent at the beginning of each trial from the microcontroller of the XBI to the parallel port of the Cereplex Direct system. The recording system saves the trial number together with a time stamp. During offline synchronization, time points for the trial starts sent by the XBI and the time points for trial starts received by the recording system are matched together with a maximal jitter of 5 ms, achieving comparable standards as in common chair-seated setups. If the animal moves away from the testing device, neural data transmission might fail because the animal is not close enough to the antennas during these times. Given that behavioral data are similarly only collected when the animal interacts with the testing device and a trigger signal is set at the beginning of each trial, temporary interruption of the neural signal does not affect the recording. This is equivalent to classical chair-seated approaches, in which intertrial periods with less constrained behavior are typically not included in the data analysis.

Before each recording session, the animals are briefly seated into the primate chair for weight measurements and implant care. The wireless head stages are positioned on the implant and secured with custom made protection cap. After the recording session, the animals are briefly seated into the primate chair again and the wireless head stages are removed from the chronic implant. Offline charging takes ∼1 h (using a USB charger) and the same set of head stages is ready to be used in another animal.

### Behavioral task design

Animals performed a visually-instructed, goal-directed, double-reach task (Fig. 1*D*). We expected spatially selective activity levels during preparation and conduction of planned reaches, as typical for the implanted brain areas (Westendorff et al., 2010; Berger et al., 2020; Taghizadeh et al., 2022). In this task, animals were required to sequentially perform two reach movements toward two targets presented on the touchscreen. Animals initiated a new trial by touching the start icon (white circle in lower part of screen, diameter: 4.8 cm). Upon trial initiation, two smaller circles (diameter: 2.4 cm) were presented next to the start icon and served as visual cues indicating to the animals the sequence of targets to reach. Simultaneously to the presentation of the visual cues, four colored target stimuli of identical size (diameter: 4.8 cm) were shown on the screen (two targets and two distractors). Figure 1*D* shows two out of four possible horizontally aligned target and distractor position in the middle and top row each. After a variable time period between 1600 and 2400 ms the start icon as well as the color cues disappeared instructing the monkey to start the movement (Go Cue). At this point, the animal was required to sequentially reach the targets that matched the visual cues (first the target indicated by the visual cue at the bottom and after that the target indicated by the top visual cue). Reaching the first target resulted in the disappearance of the touched target and the distractor on the same row. Upon reaching the second target, fluid reward was delivered to the monkey. After an intertrial interval of 1400–2600 ms, the start icon was shown again, to signal to the animal that another trial was ready to be initiated.

As with any empirical neuroscientific approach, the scope of research questions that can be addressed depend on the behavioral parameters that can be registered or controlled well in the available setup. For example, the version of the XBI presented here does not feature real-time gaze control, which could be implemented if required. Offline gaze analysis is conceivable based on the recorded facial videos provided proper calibration. Also, experiments in freely moving animals imply variable poses, which can be video-tracked if needed. Potential distractions from humans interacting with other animals in the group-housing facility can be avoided by scheduling the recording sessions for times when there is less activity/distractions present in the facility.

### Data analysis

Broadband data were preprocessed as described in Dann et al. (2016). Preprocessing steps included filtering using a sliding window median containing 91 data points (≈3 ms) and 5000-Hz low-pass filtering with a zero-phase second order Butterworth filter. In addition, we performed principal component analysis (PCA) to remove shared noise across channels per each array. The signals from each array separately were transferred into PC space. PCs that represented signals common to all channels were removed and the remaining signals were transformed back. Spike detection was done using positive or negative threshold crossing. The threshold was set individually per channel to 5.6 times the median of the absolute values of the preprocessed signal. Spikes were sorted manually using Plexon Offline Sorter V4 (Plexon Inc). Spike densities for all units were computed by convolving the spike train for each trial of a given unit with a normalized Gaussian with standard deviation of 50 ms and sampling the convolution at 200 Hz. Spiking data were analyzed aligned with relevant task events, namely the onset of the color cue, the go cue, and the hand movement onset. In the time windows surrounding these time markers, we studied the effect of target position on firing rate, as a proof-of-concept for task-specific neural response selectivity.

All calculations were performed using MATLAB 2018b. For visualization, we used the MATLAB toolbox gramm (Morel, 2018; https://github.com/piermorel/gramm).

## Results

### High task engagement and performance

We conducted electrophysiological experiments in the home environment of the animals, collecting data during 10 sessions from each animal. The sessions were recorded during working days with animal care takers, vets, and scientists entering and leaving the facility and interacting with animals in the neighboring enclosures. Despite these potential distractors, both animals showed high and consistent task engagement. Across sessions, animal K performed 963 ± 126 (mean ± SD) trials (animal H: 604 ± 201) on average with an overall performance of 91 ± 6% (mean ± SD; animal H: 72 ± 4%) per session. Reaction and movement time were comparable across sessions with a mean reaction time to go cue of animal K of 370 ± 12 ms (mean ± SD; animal H: 370 ± 8 ms) and a movement time until target acquisition of 136 ± 14 ms (mean ± SD; animal H: 107 ± 7 ms). The recording time was determined by either the session duration, i.e., until the animal stopped working, or by the time the batteries of the wireless head stages lasted, which was about 2 h.

### Simultaneous wireless recordings from three brain areas

**Figure 2.**
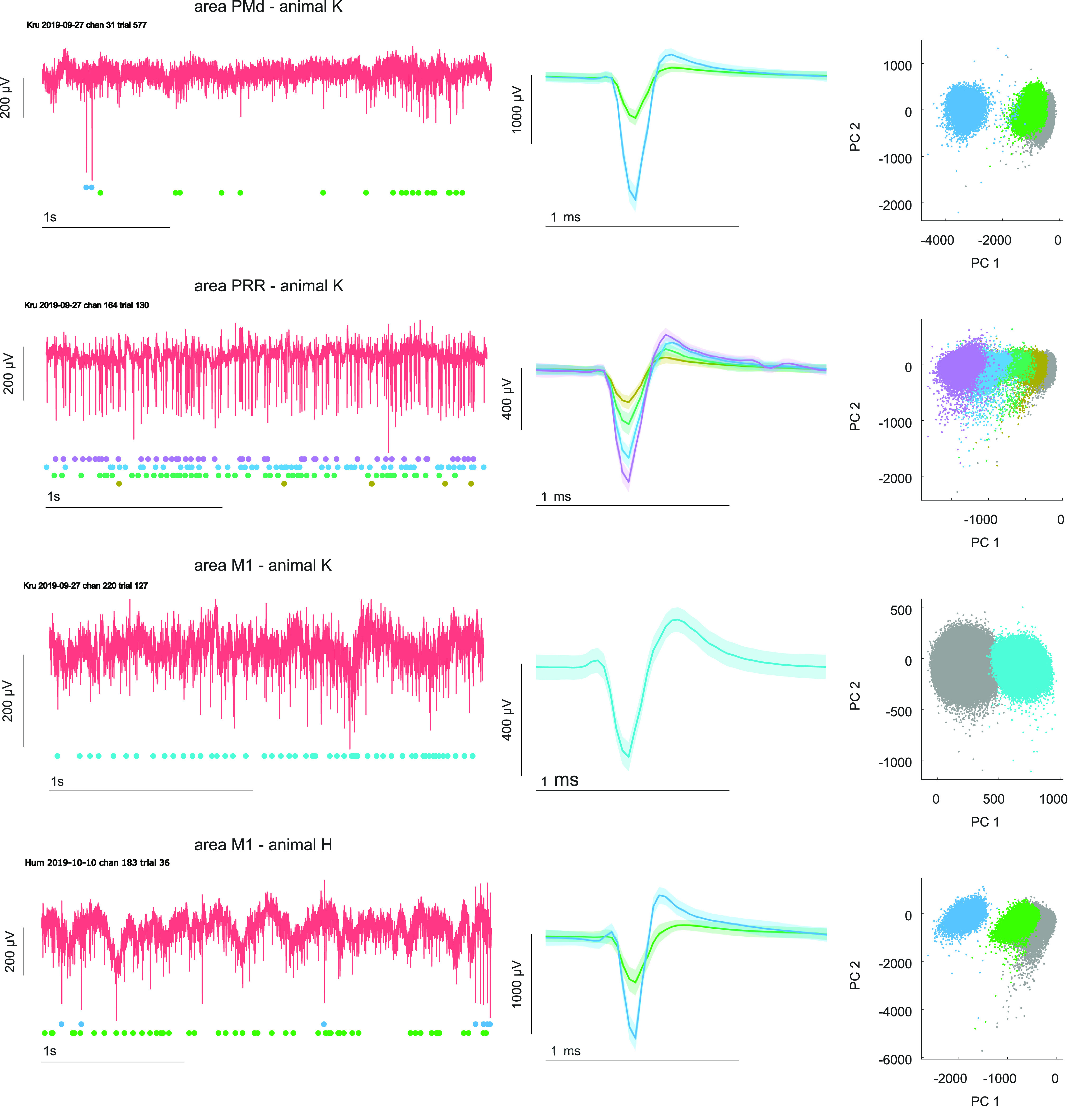
Wireless recording in home enclosure. Each row represents the neural signal from an example electrode. One electrode per recorded area is shown. Red traces show the raw data trace from one example trial. The second column shows the extracted average wave forms after preprocessing and spike sorting, based on a principal component (PC) analysis. Corresponding clusters in first two PCs are shown on the right. For visualization purposes we only show the first two PCs. For spike sorting we used additional features including third PC, nonlinear energy, and interspike interval. Colored traces below raw data represent spike trains for each sorted unit in the example trial. Shaded areas for wave forms indicate standard deviation.

The aim of the presented study was to provide a proof-of-principle for electrophysiological recordings in the home-enclosure of the animals while they were performing a cognitive task. Figure 2 displays raw data traces of an example channel and an example trial from each of the recorded areas including the extracted spike waveforms and its first two principal components from the corresponding channel. These channels were chosen to show that given a good signal recorded on the chronic arrays, the system transmits it with sufficient quality that allows for waveform separation of multiple units.

### Neural modulation dependent on reach target position

In order to assess the feasibility of the combination of our XBI with wireless neural recordings in the home environment, we instructed both animals to make movements to one out of four horizontally aligned targets and looked for neural tuning in individual cells based on the spatial position of the reach target.

**Figure 3.**
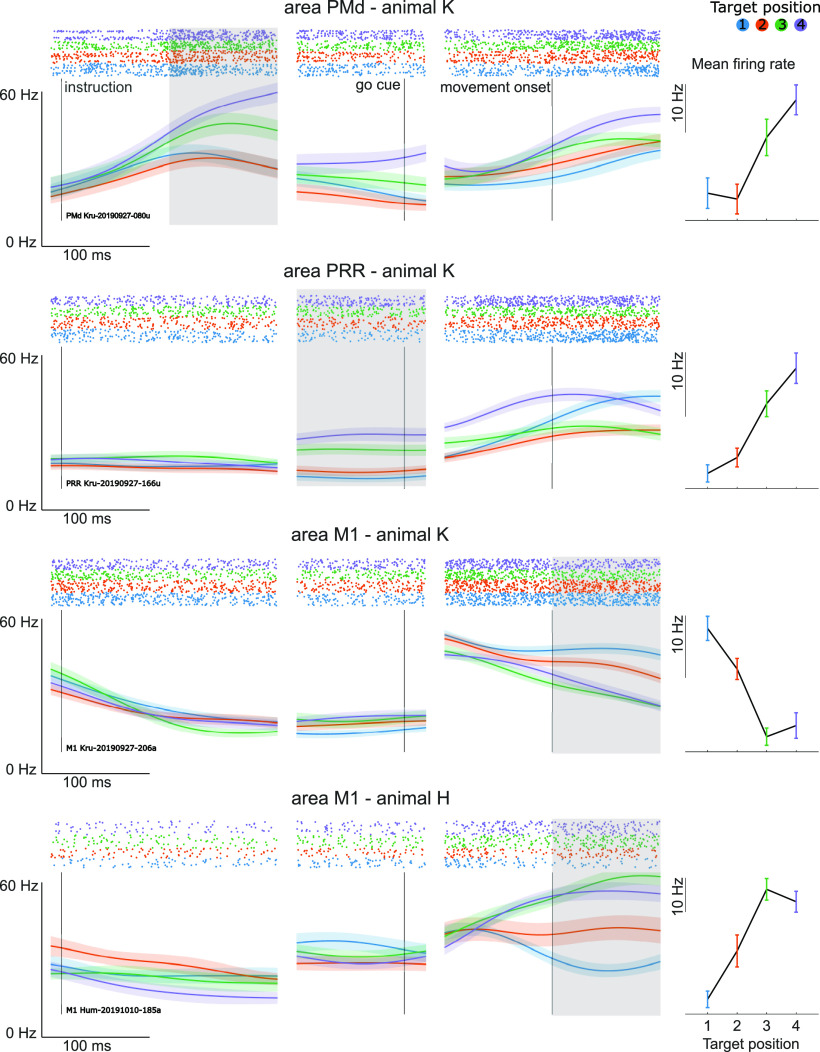
Neural selectivity for spatial task parameters. Each subfigure shows the target position dependent tuning for one example unit from each recorded area. For each unit, a raster plot and averaged spike densities are shown aligned to three different task events (instruction of reach target, go cue, movement onset). Colors correspond to four different horizontally aligned target positions (shown at the top). Shaded areas show standard error of the mean. The panel on the right shows the mean firing rate of the unit from the highlighted alignment for the four-target positions. Error bars correspond to 95% CI.

Including multiunit activity, we found 75 units to be modulated by the task structure in monkey K. Of these, 67 (89%) showed a significant (*p* < 0.05, GLM assuming Poisson distribution with four target positions as predictors) effect of target position in one of the three trial events. In monkey H we found 48 units, 37 of which (77%) showed significant modulation by target position. One example cell per recorded area is shown in Figure 3.

## Discussion

We recorded wireless neural data from two macaque monkeys performing a visually-instructed, goal-directed, double-reach task in their home enclosure in vocal and visual contact with their conspecifics. Neural and behavioral data were collected from both animals in ten sessions each, a single session lasting roughly 2 h. We collected broadband data at a single unit resolution from three different brain areas in parallel (PMd, PRR, and M1 for animal K and M1 for animal H) while the animals engaged in the behavioral task in a self-paced manner and without physical restraints. The transmission rate of 30 kHz with 16-bit amplitude resolution allows for spike sorting and multiple units could be separated on an individual channel. Selected units show tuning features based on reach target position similar to what is known from chair-seated studies.

### Transmission rate

While the presented study allowed the animals to freely move during data collection, the XBI design encourages a stable head position. We achieved a high transmission rate on all channels with on average 1.5% of dropouts. The best session in each animal stayed below 0.1% of dropout rate. This was helped by the relatively stable head position and strategic placement of the receiving antennas. In a previous study, where animals had to walk around as part of the experimental paradigm, dropout rates were 3.3% on average (Berger et al., 2020).

### Task-dependent neural modulation

Previous studies showed that individual neurons in the macaque’s fronto-parietal reach network, consisting of the dorsal premotor cortex (PMd) and the parietal reach region (PRR) as well as primary motor cortex (M1) selectively respond to one of multiple targets when a reach movement toward a target is visually instructed (Georgopoulos et al., 1982, 1986; Moran and Schwartz, 1999; Crammond and Kalaska, 2000; Cisek et al., 2003; Gail and Andersen, 2006; Klaes et al., 2011). About two third of the recorded neurons in our experiment showed target location specific modulation. In the chosen task design, the reach targets are aligned in a horizontal line, covering only one dimension of a 2D reach space on the touch screen.

If the research question requires more detailed reach kinematics, we can combine the current setup with pose estimation as previously described in Berger et al. (2020).

### Wireless recordings in the home environment

Several studies showed the possibilities to perform electrophysiological recordings in largely unrestrained macaques using untethered recordings. Yet, they mostly require the animal to be transported to a dedicated setup for behavioral and neural data collection (Foster et al., 2014; Schwarz et al., 2014; Yin et al., 2014; Capogrosso et al., 2016; Libedinsky et al., 2016; Rajangam et al., 2016; Berger et al., 2020; Milton et al., 2020; Mao et al., 2021). There have also been untethered recordings within the living enclosure of the animals. However, these untethered recordings served mainly as technical test recordings to validate the recording system during spontaneous behavior without animals performing a structured cognitive task (Jackson et al., 2006, 2007; Chestek et al., 2009; Zanos et al., 2011; Borton et al., 2013) or to study sleep (Yin et al., 2014; Xu et al., 2019; Zhou et al., 2019) without cognitive testing. The combination of our XBI with wireless recordings permits to perform neuroscientific experiments bringing cognitive tasks and neural recordings together in animals’ home enclosure. This way, animals can remain within their home environment during neural data recordings and can interact with the XBI to perform cognitive tasks in an unrestrained and self-paced manner.

### Welfare and practical considerations

System-level neurophysiological studies in awake behaving animals usually require partial separation of the animals from their social group. While a causal link between social isolation and acute stress level has not been established yet, it is reasonable to assume that temporary separation of animals from their conspecifics can negatively affect the welfare of the animal, and thus should be avoided when possible. Performing cognitive tests with in-cage devices has been shown to be not only feasible (Rumbaugh et al., 1989; Crofts et al., 1999) but has become a standard tool for cognitive testing. In some cases, the animals’ motivation to perform the task was shown to be higher in such contexts compared with the sessions where the animal was restrained in a monkey chair (Curry et al., 2017).

Compared with chair-seated approaches, the self-paced nature of cage-based approaches at a kiosk system entails that throughout the recording session the animal is provided with the opportunity to disengage from the experimental task. The animal can anytime perform other behaviors like looking or walking around, self-grooming and being in auditory and sometimes visual contact with the conspecifics, which would reduce the potential burden of chair-associated movement constraints.

In addition to the benefits for the animals, establishing routine cage-based wireless recordings from nonhuman primates provides several practical advantages as well. Being able to run unsupervised data acquisition sessions in a fully automatized fashion (Berger et al., 2018; Calapai et al., 2022) saves time and resources, with a single experimenter remotely and occasionally supervising and handling multiple devices running in parallel. This means, our approach of combining automated cognitive testing with untethered electrophysiology within the existing home environment allows upscaling the number of animals tested in parallel, thereby partially overcoming limitations in primate neuroscience that result from the need of extensive lab space with conventional methods.

Although the animal needs to be close to the recording antennas for data transfer, the system presented here can be scaled up to bigger environments. Animals can freely move within such spaces and when ready to perform the task, they move to the dedicated section where the antennas for the wireless recording and the cognitive testing system are located. Using an identification system on our XBI, as has been done in other systems (Fagot and Paleressompoulle, 2009; Butler and Kennerley, 2019; Cabrera-Moreno et al., 2022; Calapai et al., 2022), wireless recording combined with cognitive testing can even be performed in a social setting.

In conclusion, we show that wireless neural recording from cage-based settings in unrestrained rhesus macaques is feasible and reliable enough to answer neurophysiological research questions. This technique complements setup-based, chair-seated settings, when the research question does not require animals to be physically constrained and to be in an isolated testing environment.

## Synthesis

Reviewing Editor: Mark Laubach, American University Decisions are customarily a result of the Reviewing Editor and the peer reviewers coming together and discussing their recommendations until a consensus is reached. When revisions are invited, a fact-based synthesis statement explaining their decision and outlining what is needed to prepare a revision will be listed below. The following reviewer(s) agreed to reveal their identity: Jan Zimmerman, Ben Hayden.

Your manuscript was reviewed by two experts in primate neuroscience. Their reviews are given below in full. If you decide to revise and resubmit, please be sure to address all points that were raised, especially the comments from the second reviewer about the framing of your manuscript and how it relates to the study by Mao published in 2021.

Reviewer 1

The authors present a novel home enclosure based experimental design setup for non human primates that couples behavioral assessment with wireless neural recordings in unconstrained NHP.

The overarching goal achieved by the authors is fantastic and the reviewer acknowledges how difficult it is to perform the experiment the authors have performed. The claim to fame of the article lies in the home cage testing, rather than transportation to a different specifically setup environment but the reviewer wonders why this is crucial? The authors for example cite Curry et al. 2017 as a motivation but that paper clearly does not capture the breath of experimental designs performed in chairs. Furthermore, the argument is a straw man since the homecage testing itself is highly restrictive in comparison to a larger cage or dare I say the wild. I find these arguments to be overstatements and while that is of course personal it would be beneficial for the authors to tone down this rhetoric.

The article is a proof of concept technical report and is lacking crucial citations of previous work as well as details on the experiments performed including acknowledgement of the limitations.

Major points:

1. The introduction does not acknowledge the many studies that have been performed using wireless ephys in NHP, especially in the BMI community. I encourage the authors to be a bit more diligent on their citation practice.

2. The transmission rates reported in the manuscript are a bit problematic for real world usage, especially when the antennas are further away from the animal. It seems that this solution would be difficult to scale and this should be more thoroughly acknowledged.

3. How are the batteries charged? Does that not require to take the animal out of the cage system ?

4. Considering the proliferation of deep learning pose based estimation systems like DeepLabCut, SLEAP etc. it would be beneficial to discuss how this fits into the greater scheme of relating neural data and movement activity.

Reviewer 2

This is a methods paper describing the techniques used the the research group to record brain activity in rhesus macaques moving in a large cage environment. The paper includes details on the caging system, the recording system, and results of some basic data collection and analysis.

There“s nothing wrong with the paper, per se. But I think it cannot be published as it - there”s an existing published paper that uses from what I can tell are nearly identical methods. Thus, the novelty of this work is unclear. Note that this paper is not cited here, but should be:

Mao, D., Avila, E., Caziot, B., Laurens, J., Dickman, J. D., & Angelaki, D. E. (2021). Spatial modulation of hippocampal activity in freely moving macaques. Neuron, 109(21), 3521-3534.

I am not opposed to pure methods papers - that is a decision for the editor - but the framing of this paper is not justified given the existence of Mao et al. For example:

“We conclude that when movement restraints and/or a highly controlled, insulated environment are not necessary for scientific reasons, cage-based wireless neural recordings at computer-controlled kiosk systems like the XBI are a possible alternative.”

It would seem that this conclusion has already been reached. Perhaps there is some important difference between the present work and the previous one. If so, the authors need to specify the nature of their advance. The same is true of the significance statement, which would seem to indicate that the authors believe their work is the first in this domain.

There“s another major issue I would like to mention. Since this is a methods paper, its primary audience will be scholars who are attempting to do the same thing this group is doing - researchers who want instructions. Those researchers will want a few things that are lacking here.

* Reasons for why key decisions were made they way they were

* Lists of alternative approaches that would also work (presumably other researchers will have slightly different goals and constraints).

* Lists of lessons learned and mistakes not to make.

* Descriptions of tradeoffs involved in doing this kind of research

Overall, this is written like the Methods section of an empirical paper. But that is not the best way (in my view) to write a Methods paper. The methods paper has a different goal than the methods section of an empirical paper. Instead, the paper is only useful if people want to do the exact same thing as the authors have done. If they want to do the same general thing it will not be useful.
